# Draft genome sequence of *Armillaria calvescens* strain YAFA0618 associated with *Gastrodia elata*

**DOI:** 10.1128/mra.00858-24

**Published:** 2025-01-21

**Authors:** Gaosheng Wang, Yumin Zeng, Yuwu Bu, Xiaolong Yuan, Mingjun Peng, Qianchi Zhao, Yi Wang

**Affiliations:** 1 Yunnan Key Laboratory of Biodiversity of Gaoligong Mountain, Yunnan Academy of Forestry and Grassland 74571 , Kunming, China; 2 Yunnan Forestry and Grassland Technology Extension Station, Kunming, China; 3 Yuxi Yupu Biotechnology Development Co., LTD., Tonghai, China; Rochester Institute of Technology, Rochester, New York, USA

**Keywords:** *Armillaria calvescens*, genome, Illumina sequencing

## Abstract

*Armillaria calvescens* is a symbiotic fungus of *Gastrodia elata*. Here, we present a draft genome sequence of *A. calvescens* strain YAFA0618. The genome resource will support subsequent research into the relationship between *A. calvescens* and *G. elata*.

## ANNOUNCEMENT

The genus *Armillaria* (Fr.: Fr.) Staude (Physalacriaceae, Agaricales, Basidiomycota) are well known as important plant root-rot pathogens (1, 2). *Armillaria calvescens* and other species of *Armillaria* have been identified as symbionts of *Gastrodia elata* Bl. and *Polyporus umbellatus* (Pers.) Fr (3, 4). Both the rhizome of *G. elata* and *P. umbellatus* are important traditional medicine in East Asia (5, 6). Some species of *Armillaria* also are edible and medicinal lignicolous basidiomycete, such as *A. mellea* (7). In this study, we report a draft genome sequence of *A. calvescens* associated with *G. elata*.

The fungus *A. calvescens* strain YAFA0618 was isolated from the tuber of wild G. *elata*. The isolation process was as follows. The tuber of *G. elata* was collected from Gaoligong Mountain, Yunnan Province, China (25°17′24″ N, 99°18′27″ E) in 2022. The tuber of G. *elata* was washed by soaking it in tap water for 6 hours and then rinsing it with sterile water. A tissue grinder was used to grind the tuber, after which sterile water was added to obtain a slurry. Finally, the tuber slurry was further diluted and spread onto a solid malt-yeast medium (Difco, Lawrence, KS, USA) containing 200 µg/mL of kanamycin and streptomycin, respectively. After 10 days of incubation (28°C), a single colony was transferred to a fresh malt-yeast medium. One single colony (strain YAFA0618) was identified by sequencing the ITS (Internal Transcribed Spacer) region with ITS1F and ITS4 amplification as belonging to *A. calvescens*, and the ITS sequence was deposited in GenBank (accession number PQ060156).

The mycelia of *A. calvescens* YAFA0618 were harvested for DNA extraction after cultivation in malt-yeast liquid medium at 28°C for 15 days on a rotary shaker at 150 rpm. Genomic DNA was extracted using a DNeasy minikit (Qiagen, Valencia, CA, USA) following the manufacturer’s instructions. Illumina (San Diego, CA, USA) sequencing libraries were prepared using a TruSeq DNA Sample Prep Kit (Illumina) with insert sizes of 400 bp. The library was sequenced on an Illumina NovaSeq 6000 platform with a 2 × 150 bp paired-end configuration. A total of 69,585,692 raw reads were obtained (Table 1). High-quality cleaned sequences (10.12 Gb; coverage, 130×) were obtained by trimming the adapter sequences with AdapterRemoval version 2 (8). The *de novo* genome assembly was conducted using SPADes v. Nov-2021 (9). The default parameters were used for all the software unless otherwise specified. The genome assembly size of the YAFA0618 strain was 92,249,651 bp with 74 scaffolds. The longest scaffold was 5,351,494 bp, with an N50 value of 1,906,071 bp and an N90 value of 661,081 bp. The GC content was 47.53%. The completeness of the genome assembly was assessed using BUSCO version 5.2.2 (10) with the OrthoDBfungi v10 (fungi_odb10) database, and its completeness value was 98.2%.

**TABLE 1:** Genome sequencing statistics

Parameter	Value
Raw reads	
Reads number	69,585,692
Total bases (bp)	10,122,729,575
Coverage	110×
Genome assembly	
Total base	92,249,651
Total scaffolds	74
Max sequence length	5,351,494 bp
N50	1,906,071 bp
N90	661,081 bp
GC_content (%)	47.50
Genome assembly assessment	
Complete BUSCOs	98.2%
Complete and single-copy BUSCOs	94.9%
Complete and duplicated BUSCOs	3.3%

## Data Availability

The raw reads for genome sequencing have been deposited in the NCBI Sequence Read Archive under accession number SRR29913577. The draft genome sequence of Armillaria calvescens YAFA0618 was deposited in the DDBJ/ENA/GenBank database under accession number JBFTYQ000000000 (BioProject/BioSample PRJNA1138632/SAMN42723733).
